# Exploring the Optimal Strategy to Predict Essential Genes in Microbes

**DOI:** 10.3390/biom2010001

**Published:** 2011-12-26

**Authors:** Jingyuan Deng, Lirong Tan, Xiaodong Lin, Yao Lu, Long J. Lu

**Affiliations:** 1 Division of Biomedical Informatics, Cincinnati Children’s Hospital Research Foundation, 3333 Burnet Avenue, Cincinnati, OH 45229-3026, USA; Email: dengjn@gmail.com (J.D.); lrtan.lydia@gmail.com (L.T.); 2 Department of Computer Science, School of Computing Sciences and Informatics, University of Cincinnati, 814 Rhodes Hall, Cincinnati, OH 45221-0030, USA; 3 Department of Environmental Health, College of Medicine, University of Cincinnati, 231 Albert Sabin Way, Cincinnati, OH 45267-0524, USA; 4 Department of Management Science & Information Systems, Rutgers University, 252 Janice H. Levin Hall, Piscataway, NJ 08854, USA; Email: xiaodonglin@gmail.com; 5 Shanghai Institute of Medical Genetics, Shanghai Jiaotong University, 24/1400 Beijing (W) Road, Shanghai 200040, China; Email: lvyao2005@hotmail.com

**Keywords:** essential genes, machine learning, annotation

## Abstract

Accurately predicting essential genes is important in many aspects of biology, medicine and bioengineering. In previous research, we have developed a machine learning based integrative algorithm to predict essential genes in bacterial species. This algorithm lends itself to two approaches for predicting essential genes: learning the traits from known essential genes in the target organism, or transferring essential gene annotations from a closely related model organism. However, for an understudied microbe, each approach has its potential limitations. The first is constricted by the often small number of known essential genes. The second is limited by the availability of model organisms and by evolutionary distance. In this study, we aim to determine the optimal strategy for predicting essential genes by examining four microbes with well-characterized essential genes. Our results suggest that, unless the known essential genes are few, learning from the known essential genes in the target organism usually outperforms transferring essential gene annotations from a related model organism. In fact, the required number of known essential genes is surprisingly small to make accurate predictions. In prokaryotes, when the number of known essential genes is greater than 2% of total genes, this approach already comes close to its optimal performance. In eukaryotes, achieving the same best performance requires over 4% of total genes, reflecting the increased complexity of eukaryotic organisms. Combining the two approaches resulted in an increased performance when the known essential genes are few. Our investigation thus provides key information on accurately predicting essential genes and will greatly facilitate annotations of microbial genomes.

## 1.Introduction

Essential genes are defined as those that, when disrupted, confer a lethal phenotype to microorganisms under defined conditions. As such, the essentiality of a gene is the indispensability of this gene’s product to the survival of a microorganism. A complete understanding of gene essentiality is important in multiple facets of biology, medicine and bioengineering. For example, because of the lethal consequences of their disruption, essential genes are often attractive targets of antibiotics [1]. Essential genes of an organism also constitute its minimal gene set, a key concept in the emerging field of synthetic biology [2,3]. Furthermore, studying gene essentiality is a crucial step toward unraveling the complex relationship between genotype and phenotype [4], a fundamental question in genetics. 

Systematic genome-wide interrogations of essential genes have been conducted by single gene knockouts [5,6,7,8], transposon mutagenesis [9,10,11,12,13,14,15], or antisense RNA inhibitions [16,17]. Although the efficiency of gene deletion has improved, performing large-scale experiments to knock out each gene encoded in an organism’s genome, usually in the magnitude of thousands, is still a daunting task. The work of experimentally identifying essential genes in an organism is even more formidable than was once thought as researchers have found that growth conditions can significantly alter the spectrum of essentiality in bacteria [18,19,20,21,22] and yeast [23]. Therefore, computational methods for predicting essential genes become an appealing option for circumventing the expense and difficulty of experimental screens. A computational prediction is especially useful when the organism is either unculturable, such as *Pneumocystis carinii*, or difficult to perform gene disruption on, such as *Aspergillus fumigatus*.

In our previous research, we developed a machine-learning based algorithm that predicts essential genes by integrating diverse types of information encoded in a microorganism’s genome that are potentially associated with gene essentiality [24]. We tested this algorithm in four bacterial species whose essential genes have been well characterized: *Escherichia coli* (*EC*), *Pseudomonas aeruginosa* (*PA*), *Acinetobacter baylyi* (*AB*) and *Bacillus subtilis* (*BS*). Ten-fold cross-validations in each organism showed a high predictive accuracy (AUC: ~0.9). We also reported that gene essentiality can be reliably transferred using features trained and tested in a distantly related microorganism (AUC: 0.69–0.89). Cross-organism predictions significantly outperformed homology mapping. 

Our algorithm thus significantly extended our ability to predict essential genes beyond orthologs by providing two alternative approaches: We can learn the characteristics underlying the subset of known essential genes in one organism and predict the essentiality of the rest of the genes in the same organism. Alternatively, we can transfer the gene essentiality from its most closely related model organisms where a complete set of essential genes is available. However, to determine the essential gene set in an understudied microbe, both approaches have potential limitations. The first approach is limited by the often low number of known essential genes, while the second approach is limited by the availability of model organisms and the evolutionary distance to the target organism. Although our previous work demonstrated that both approaches are capable of producing accurate predictions, further study is needed to determine the most suitable situation each approach can be employed.

The current study represents a significant progress since our previous work by aiming to determine an optimal strategy for predicting essential genes in an understudied microbe by examining these potential limitations with regard to the above-mentioned approaches and a third approach that combines the two approaches. We performed our investigations on two pairs of microbes with well-characterized essential genes: two prokaryotes, *Escherichia coli* K-12 (*EC*) and *Acinetobacter baylyi* ADP1 (*AB*) and two eukaryotes, *Saccharomyces cerevisiae* S288c (*SC*) and *Neurospora crassa* OR74A (*NC*). We withheld different fractions of known essential genes in each organism and evaluated the predictive performance. Through these simulations, we were able to reveal the conditions under which each approach is most suitable for predicting essential genes in a microbe with respect to the size of known essential genes. The results obtained from our study will greatly facilitate the annotations of microbial genomes and provide valuable information to synthetic biology. 

## 2.Experimental

### 2.1. Data Sources

*E. coli* K-12 sequence data were downloaded from Comprehensive Microbial Resource (CMR) database at http://cmr.jcvi.org. It contains 4289 protein sequences in total [25]. The essential genes of *E. coli* K-12 were downloaded from the PEC database [7]. The Kato dataset contains 302 essential genes from gene deletion experiments. 

*A. baylyi ADP1* sequences were collected from the Magnifying Genomes database (http://www.genoscope.cns.fr/agc/mage). Of a total of 3308 genes, 499 are essential genes from de Berardinis *et al*. [6]

*S. cerevisiae S288c* sequences were downloaded from Saccharomyces Genome Database at: http://downloads.yeastgenome.org/sequence/genomic_sequence/. It contains 5885 ORFs. The essential gene list was downloaded from Giaever *et al*. [26]. This dataset contains 1049 essential genes from targeted mutagenesis experiments. 

*N. crassa OR74A* ORFs were downloaded from *Neurospora crassa* database at Broad Institute at http://www.broadinstitute.org/annotation/genome/neurospora/MultiDownloads.html. Dubious ORFs and pseudogenes were excluded from this list. The essential gene dataset was kindly provided by K. Borkovich at UC Riverside from the systematic genome deletion project in *N. crassa*. This list contains 7172 experimental verified essential/nonessential genes, and 1251 of them are essential genes.

Gene expression data in these organisms were downloaded from NCBI GEO [27], ArrayExpress [28], and the gene-expression profiles of microarray data from Gasch *et al*. [29].

### 2.2. Genomic Features

Based on our previous research, we considered three main types of features: (A) those intrinsic to a gene’s sequence (e.g., GC content, length); (B) those derived from genomic sequence (e.g., localization signals and codon adaptation measures); and (C) experimental functional genomics data (e.g., gene-expression microarray data) (Table 1). The detailed descriptions of these features and their biological implications can be found in the supplemental materials as well as in Deng *et al*. [24]. For example, domain enrichment score (DES) reflects the conservation of local domains rather than the entire gene, which is calculated by the ratio of the domain’s occurrence frequencies in essential genes *vs.* in total genes in a given organism. In another example, phylogenetic score (PHYS) measures the evolutionary conservation of a gene, which is calculated by counting the number of genomes that have orthologous hits. Such conservation has been shown to correlate well with the indispensability of a gene. These diverse types of features suggest that gene essentiality is likely determined not solely by the genomic sequence, but by multiple aspects of biology coinciding. 

**Table 1:** Thirty-five considered features.

Feature		Description		Class *	Data type	Available **
Aromo		Aromaticity score		A	Real	**EC**/**AB**/**SC**/NC
A3s		Base composition A		A	Real	EC/AB/SC/NC
C3s		Base composition C		A	Real	EC/AB/SC/**NC**
G3s		Base composition G		A	Real	EC/**AB**/SC/NC
T3s		Base composition T		A	Real	EC/**AB**/SC/**NC**
CAI		Codon adaptation index		A	Real	EC/**AB**/SC/**NC**
CBI		Codon bias index		A	Real	**EC**/AB/SC/NC
Fop		Frequency of optimal codons		A	Real	EC/AB/**SC**/NC
Nc		Effective number of codons		A	Real	**EC**/**AB**/**SC**/**NC**
L_sym		Frequency of synonymous codons		A	Integer	EC/AB/SC/NC
L_aa		Length amino acids		A	Integer	**EC**/**AB**/**SC**/**NC**
GC		GC content		A	Real	EC/AB/**SC**/**NC**
GC3s		GC content 3rd position of synonymous codons		A	Real	EC/AB/SC/NC
Gravy		Hydrophobicity score		A	Real	EC/AB/**SC**/NC
Cytoplasm		Subcellular localization: cytoplasm		B	Boolean	**EC**/**AB**/**SC**/NC
Extracellular		Subcellular localization: Extracellular		B	Boolean	**EC**/AB/**SC**/**NC**
Inner		Subcellular localization: Inner membrane		B	Boolean	**EC**/**AB**
Outer		Subcellular localization: Outer membrane		B	Boolean	EC/AB
Periplasm		Subcellular localization: Periplasm		B	Boolean	EC/AB
Golgi		Subcellular localization: Golgi		B	Boolean	SC/NC
Nucleus		Subcellular localization: Nucleus		B	Boolean	**SC**/**NC**
Mito		Subcellular localization: Mitochondrion		B	Boolean	SC/NC
Plasma		Subcellular localization: Plasma membrane		B	Boolean	SC/**NC**
Vacuole		Subcellular localization: Vacuole		B	Boolean	SC/NC
Peroxisome		Subcellular localization: Peroxisome		B	Boolean	SC/NC
ER		Subcellular localization: Endoplasmic reticulum		B	Boolean	SC/NC
ExpAA		Expect number of Amino acids in helices		B	Real	EC/AB/SC/NC
First60		Expect number of AAs in helices in first 60 AAs		B	Real	EC/AB/SC/NC
PredHel		Number of predicted TM helices		B	Integer	EC/AB/**SC**/**NC**
PHYS		Phylogenetic score		B	Real	**EC**/**AB**/**SC**/**NC**
PA		Paralogy		B	Boolean	**EC**/**AB**/**SC**/**NC**
DES		Domain enrichment score		B	Real	**EC**/**AB**/**SC**/**NC**
FLU		Fluctuation		C	Real	**EC**/**SC**/**NC**
CEH		Coexpression network hubs		C	Boolean	**EC**/SC/NC
CEB		Coexpression network bottlenecks		C	Boolean	**EC**/SC/NC

*—Class A: Sequence-based intrinsic features; Class B: Sequence-derived intrinsic features; Class C: Context-dependent features; **—Features used in the training and testing in each organism are in bold.

We evaluated these features based on their predictive power following a procedure described in Deng *et al*. [24]. To briefly summarize, we performed a logistic regression analysis and ranked all features according to the cover length of log-odds ratio. A longer overall coverage length indicates greater contribution of the corresponding feature to the gene essentiality. Because we were more interested in predicting essential genes rather than non-essential genes, the features with a positive coverage length were our candidate features. We also considered prior biological information to remove feature redundancy. 

### 2.3. Training and Testing Sets Preparation

The training data included the attribute values for each feature and the class assignments. Each gene was assigned a Boolean value regarding its essentiality (1—essential; 0—non-essential). The training data were divided into 10 equal parts. Nine parts were used to train the classifiers and the remaining part was used for testing. The control training set was generated by randomly assigning essential labels to all genes. The same number of random “essential genes” as the number of true essential genes was used in the training and testing frame.

#### 2.3.1. Same-Organism Approach

For each of the four organisms (*i.e.*, *EC*, *AB*, *SC* and *NC*), we withheld different fractions of known essential genes to simulate the situation that only partial true essential genes were known. These known essential genes were selected through random sampling and comprised of our “gold standard” positive set. Because there are more non-essential genes than essential genes (10:1 in prokaryotes and 5:1 in eukaryotes), we constructed our training datasets with the same essential *vs.* non-essential ratio to resemble the situation in nature. That is, for a “gold standard” positive set of size *N*, we randomly selected *xN* (*x =* 10 for prokaryotes, and 5 for eukaryotes) genes from the non-essential genes as the “gold standard” negative set. We then solved the problem of imbalanced training set through data re-sampling, where we extracted a smaller set of non-essential genes while preserving all the essential instances. This method modifies the prior probability of the non-essential and essential classes to obtain a more balanced training set. Similar approaches have been used in other studies [30,31]. We trained our model using this training set. Each time we repeated the random process 200 times to obtain a reliable result. 

#### 2.3.2. Cross-Organism Approach

As described in Deng *et al.* [24], when predicting essential genes in each of the four organisms, the training set is the complete gene set of its paired organism. For example, when we predict essential genes in *EC*, the training set is the complete gene set in *AB*, where the complete *AB* essential genes compose the “gold standard” positive set and the remaining *AB* non-essential genes consist of the “gold standard” negative set.

#### 2.3.3. The Combined Approach

For each of the four organisms, the training set was constructed as the combination of the training sets in the same-organism approach and cross-organism approach. Meanwhile, we assigned different weights to each model organism based on the evolutionary distance to the target organism. For example, when we predicted essential genes in *EC*, the “gold standard” positive set consisted of a randomly selected fraction of essential genes in *EC* together with the complete set of essential genes in *AB*, where genes from *EC* were assigned weights *w* (*w* > 1), and those from *AB* were assigned a weight of 1. Similarly, the “gold standard” negative set consisted of the same fraction of randomly selected non-essential genes from *EC* together with the complete set of non-essential genes in *AB*, with weights *w* and 1 respectively.

### 2.4. Classifier Design

We used a logistic regression classifier to train and test the model. All classifiers were implemented using the Orange software package (http://www.ailab.si/orange/). To train and test our classifier, features were first extracted where available for each ORF and annotated with known essentiality values, thereby creating our “gold standard” data set. Then the “gold standard” dataset was divided into 10 equal parts. Nine parts were used to train the classifiers and the remaining part was used for testing.

Then we applied the model to the target organism, and predicted the probability of essentiality for each gene in that organism. Based on the true gene labels and the predicted probability, we were able to calculate the AUC (Area Under Curve) of the Receiving Operation Curve (ROC) and the Sensitivity (number of correctly predicted essential genes/total essential genes) of the prediction. AUC and Sensitivity were then used to evaluate the performance of the model.

## 3.Results and Discussion

### 3.1. Optimal Strategy for Predicting Essential Genes in EC

*EC* is a gram-negative bacterium commonly found in the lower intestine of warm-blooded organisms. It is one of the most well-studied prokaryotic model organisms and has the best-characterized essential genes. 

We compared three approaches using our previously developed integrative algorithm (Table 2): (1) the same-organism approach, where we learned traits among the partially known essential genes in *EC* and predicted the rest of the essential genes; (2) the cross-organism approach, in which we learned traits among the known essential genes in *AB*, a closely-related model organism, and tried to predict the essential genes in *EC*; and (3) the combined approach, in which we learned traits among the known essential genes in *AB* as well as the partially known essential genes in *EC* and tried to predict the rest of the essential genes in *EC*. Because in our previous research we have shown that our cross-organism approach outperforms homology mapping [24], we did not compare homology mapping in this study.

**Table 2:** Summary of the three approaches (see Experimental Section for details).

Approach	Description	“Gold Standard” Set		Prediction Set
		Training Set	Testing Set	
Same-organism approach	Learning from the limited number of known essential genes in the target organism	9/10 of the “gold standard” set of the target organism	1/10 of the “gold standard” set of the target organism	The entire set of genes except the “gold standard” in the target organism
Cross-organism approach	Learning from essential genes from a closely-related model organism	9/10 of the “gold standard” set in the related model organism	1/10 of the “gold standard” set in the related model organism	The entire set of genes except the “gold standard” in the target organism
Combined approach	Learning from known essential genes in the target organism as well as a closely-related model organism with higher weights to the former	9/10 of the “gold standard” combined set. The weights assigned to the genes in the target and model organism is w:1	1/10 of the “gold standard” combined set	The entire set of genes except the “gold standard” in the target organism

#### 3.1.1. Same-Organism Approach: Learning Traits from the Partially Known Essential Genes in *EC*

Among the total characteristic features that we considered, we have identified 13 that are potentially associated with gene essentiality in EC with relatively weak correlations among themselves (Table 1). Among these 13 features, we previously identified the domain enrichment score (DES) as the strongest [24], suggesting that gene essentiality is likely preserved through the function of protein domains or domain combinations rather than through the conservation of the entire genes. To show its efficiency, in our model construction process, we separated this dominant feature from the remaining 12 features. First, we used 12 features excluding DES to build the “no-DES” model. Next, we compiled the DES feature with the other features to form the “with-DES” model. 

**Figure 1:**
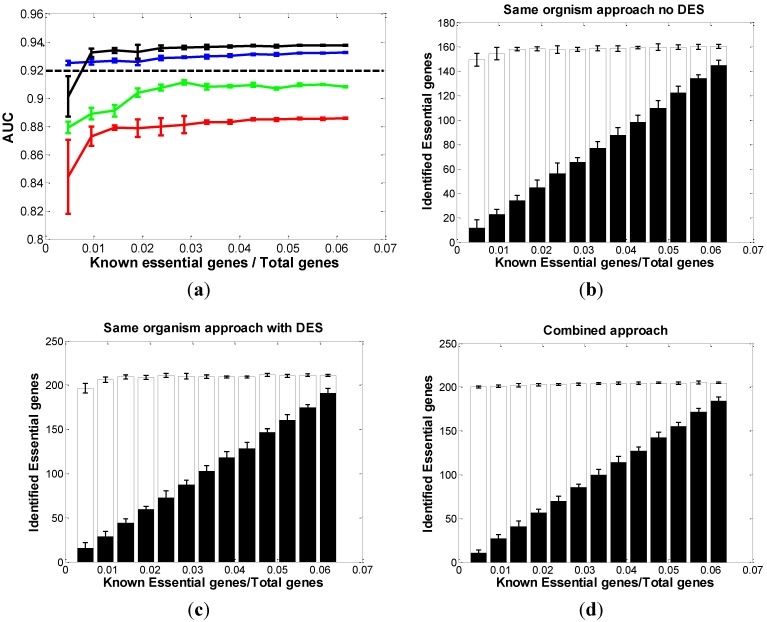
Comparison of three approaches in *EC*. (**a**) The distribution of AUC along with the different sizes of known essential genes in *EC*: red curve: same-organism approach “with no-DES”; black curve: same-organism approach “with DES”; blue curve: combined approach; green curve: the DES feature only dashed line: cross-organism approach. The bar chart of the correctly classified essential genes among the top 400 predictions with respect to the different sizes of known essential genes in *EC* using (**b**) “no-DES” model; (**c**) “with-DES” model; and (**d**) combined model. The black bar shows the correctly classified essential genes in the “gold standard” set.

We first built the “no-DES” model in *EC* (see Experimental Section). The 12 selected features were used as input variables in the logistic regression classifier. The classifier generated a probability score of essentiality for each gene of the entire target organism (both “gold standard” set and prediction set (Table 2)). Combining this probability score and the true essentiality information of each gene, we generated the ROC curve. The ROC was then evaluated by the AUC score. We gradually increased the size of known essential genes in our model. The result showed that the AUC score increased from 0.84 to 0.88 before the size of known essential genes reached 2% of the total genes in the genome. At this point, the model had already performed very closely to its optimal, achieving over 95% of its best performance. Beyond this point, the AUC score increased slowly from 0.88 to 0.89 even with a substantial increase of known essential genes (Figure 1a, red curve). 

Besides the AUC score, we were also interested in the number of genes we successfully classified. Using 10% as a cutoff, the top 400 genes with the largest probability scores were predicted as essential genes. Those 400 genes came from two parts, the “gold standard” set (Figure 1b, black bar) and the prediction set (Figure 1b, white bar). Figure 1b showed that the performance was nearly stable if the known essential genes took up more than 2% of the total genes in *EC*. 

Next, we compiled the DES feature with the other 12 features and built model by the same process used for the “no-DES” set. Compared with the “no-DES” model, the results were significantly improved (Figure 1a, black curves and Figure 1c). We can see that the AUC reached 0.94 if we knew about 2% of total genes to be essential. Figure 1c also suggested that the performance of the classification is stable if more than 2% of the total genes are known to be essential. They both decrease quickly as less essential information is given. We also applied our model only using the DES feature and compared the predictions with both the “no-DES” and “with-DES” sets (Figure 1a). The comparison showed that the DES alone is not enough to make optimal predictions, suggesting that including more features is necessary to achieve the optimal prediction performance. 

#### 3.1.2. Cross-Organism Approach: Transferring Essential Gene Annotations from *AB*

*AB* is a gram-negative bacterium commonly found in aquatic and soil environments. It belongs to the same class of gram-negative proteobacteria as *EC*. A set of 499 *AB* essential genes has been identified by targeted mutagenesis. We were able to use *AB* essential genes set to predict essential genes in *EC* [24], and the direct prediction yielded an ROC with the AUC score of 0.92. In Figure 1a, the dashed line shows the AUC of the prediction from *AB*, and the black curve dominates it when 1.5% of the total genes are known to be essential. This suggested that knowing 1.5% or more genes of the total genes to be essential in *EC* is sufficient to achieve a prediction better than transferring annotations from *AB*.

#### 3.1.3. Combined Approach: Combining Both *AB* and Partially Known Essential Information in *EC*

Based on the above results, we had a new question: If we combine both *AB* and the fraction of known genes with essential information in *EC* as the new “gold standard” set and try to predict the rest of the essential genes in *EC*, could the result be significantly improved? To answer this question, we randomly chose a fraction of genes (we gradually increased the number of known genes from 10% to 90%) from *EC* and combined them with *AB* dataset (see Experimental Section). In the model training process, we assigned different weights to the two gene sets to obtain a more reliable result. Here, the partially known genes with essential information from *EC* have been set to have 4:1 weights *vs.* the *AB* genes. We trained the model on this combined “gold standard” set. Each time we also repeated the random process 200 times to estimate the variance. The results (Figure 1a, blue curve) showed that the combined approach outperformed the same-organism approach at the beginning. However, the black curve quickly outperformed the blue curve as the known essential genes in *EC* increased. The correctly predicted genes in Figure 1d also supported this result.

### 3.2. Optimal Strategy for Predicting Essential Genes in AB

**Figure 2:**
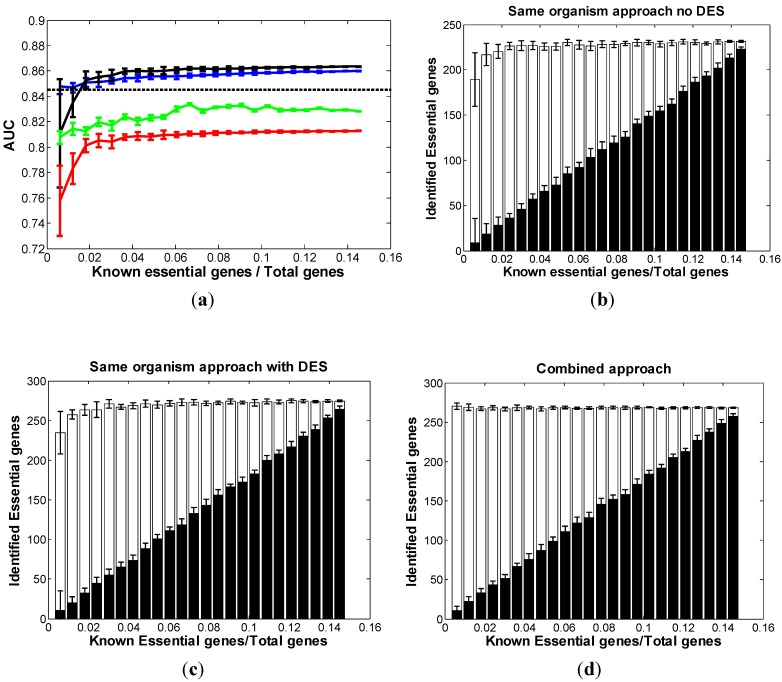
Comparison of three approaches in *AB.* (**a**) The distribution of AUC along with the different sizes of known essential genes in *AB*: red curve: same-organism approach “with no-DES”; black curve: same-organism approach “with DES”; blue curve: combined approach; dashed line: cross-organism approach. The bar chart of the correctly classified essential genes among the top 400 predictions with respect to the different sizes of known essential genes in *AB* using (**b**) “no-DES” model; (**c**) “with-DES” model; and (**d**) combined model. The black bar shows the correctly classified essential genes in the “gold standard” set.

In *AB*, we identified 11 features that are potentially associated with gene essentiality and have relatively weak correlations among themselves [24] (Table 1). We followed the same analysis procedure as in *EC*. In the same-organism approach, we first used 10 features excluding DES as the input of the classifier to build the “no-DES” model, and then including DES to build the “with-DES” model. The model generated a probability score of gene essentiality for each gene of the entire target organism. Combining this probability score and the true essentiality information of each gene, we were able to evaluate the performance. In Figure 2a, the red and black curves showed the distribution of the AUC scores of the results output from the “no-DES” and “with-DES” models respectively. Both curves increase rapidly before 2% (66/3308) of total genes are known to be essential, achieving more than 95% of the best performance. Compared with “no-DES” results, the results of “with-DES” were significantly improved. Also, the dashed line in Figure 2a shows the AUC of the cross-organism approach using *EC* essential genes, suggesting that knowing 2% of total genes to be essential is “sufficient” to lead to a prediction better than transfer from *EC*. Figure 2b and c show the bar charts of the correctly classified essential genes using the “no-DES” and “with-DES” models respectively. For *AB*, we adopted a similar percentage as the cutoff to predict essential genes as in *EC*, and the top 400 genes with the largest probability scores were predicted as essential genes. In both Figure 2b and 2c, the performance is nearly stable if the known essential genes take up more than 2% of the total genes in *AB.* In the combined approach, we combined both the *EC* essential genes with increasing numbers of known *AB* essential genes by assigning different weights. The blue curve (Figure 2a) shows the combined approach outperforming the same-organism approach only at the beginning. Compared with Figure 1, the difference between the combined approach and the same-organism approach in *AB* was less significant than in *EC*. The green curve in Figure 2a shows the performance of DES feature only. This suggests that the integration of different features is able to make more accurate predictions than using DES alone. 

### 3.3. Optimal Strategy for Predicting Essential Genes in SC

Our results suggested that essential genes are highly predictable by learning the characteristics underlying gene essentiality in prokaryotes. To test whether the same trend can also be observed in eukaryotic species, we chose *SC* and *NC* as our test candidate species. 

**Figure 3:**
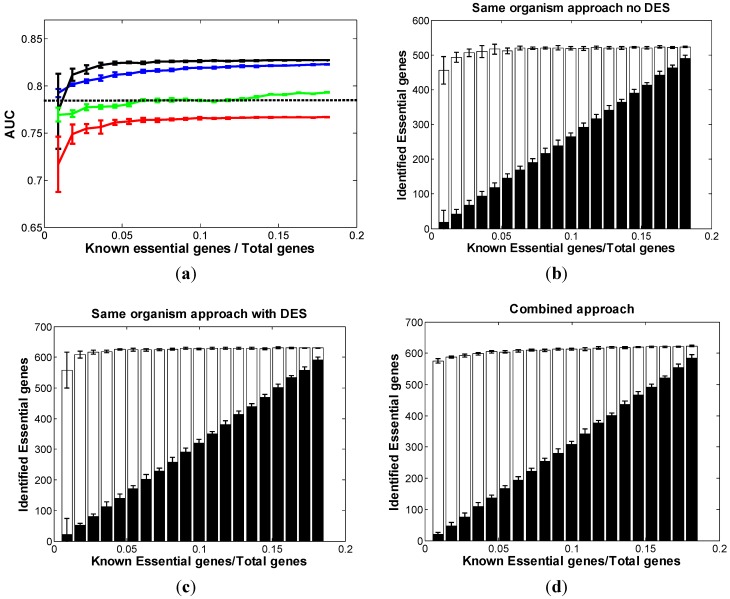
Comparison of three approaches in *SC.* (**a**) The distribution of AUC along with the different sizes of known essential genes in *SC*: red curve: same-organism approach “with no-DES”; black curve: same-organism approach “with DES”; blue curve: combined approach; dashed line: cross-organism approach. The bar chart of the correctly classified essential genes among the top 1200 predictions with respect to the different sizes of known essential genes in *SC* using (**b**) “no-DES” model; (**c**) “with-DES” model; and (**d**) combined model. The black bar shows the correctly classified essential genes in the “gold standard” set.

*SC* is an important eukaryotic model organism in cell biology and is one of the most thoroughly studied eukaryotic microorganisms. There are 1049 essential genes identified by the systematic deletion project [26]. Using the same-organism approach in *SC*, we identified 14 features potentially associated with gene essentiality (Table 1). Domain enrichment score (DES) was found to be a strong feature in predicting essential genes in eukaryotes as well. This suggests that, much as in prokaryotes, gene essentiality in eukaryotes is likely preserved through the function of protein domains or domain combinations rather than through the conservation of entire genes. First, we used 13 features excluding DES as the input of the classifier. After the 10-fold cross-validation, each gene of the target organism received a probability score of essentiality. Combining this probability score and the true essentiality information of each gene, we were able to evaluate the performance. Figure 3a (red curve) showed the AUC curve of the “no-DES” results. It gradually increases along with the increase of the known essential genes and reaches stable at around 4% point on the x-axis, achieving 95% of the best performance. Besides the AUC curve, we also plotted the bar chart of correctly predicted essential genes (Figure 3b). Since essential genes comprise of about 20% of a eukaryotic genome, we used 1200 as the cutoff, *i.e.*, the 1200 genes with the highest essential scores were predicted as *SC* essential genes. The performance increased as we increased the size of the training dataset, and the saturation point was at 4%. Figure 3a (green curve) shows that, similar to in prokaryotes, DES is a strong feature to the prediction of gene essentiality and incorporating it with other functional and genomics features is able to achieve an optimal performance. 

Next, we added the DES feature into the model. Figure 3a (black curve) and Figure 3c show a similar trend, except the values are significantly higher than those of the “no-DES” results. This further supports the notion that the DES feature has strong power in predicting essential genes in eukaryotic species. Moreover, we note that the saturation occurred at 4% point in both figures. Thus, knowing 4% or more of the total genes is essential to building a reliable prediction. 

In the combined approach, we used both *NC* and the partially known essential genes in *SC* as the new training set. Would the result be significantly improved again? We followed the same scheme as described above. The results were consistent: As shown in Figure 3a, the performance of the same-organism approach (black curve) dominates the performance of the combined approach (blue curve) from about 1.5% on the *x*-axis. Although the saturation point of the prediction is different, the dominating points are almost the same as those in *EC* and *AB*. 

### 3.4. Optimal Strategy for Predicting Essential Genes in NC

**Figure 4:**
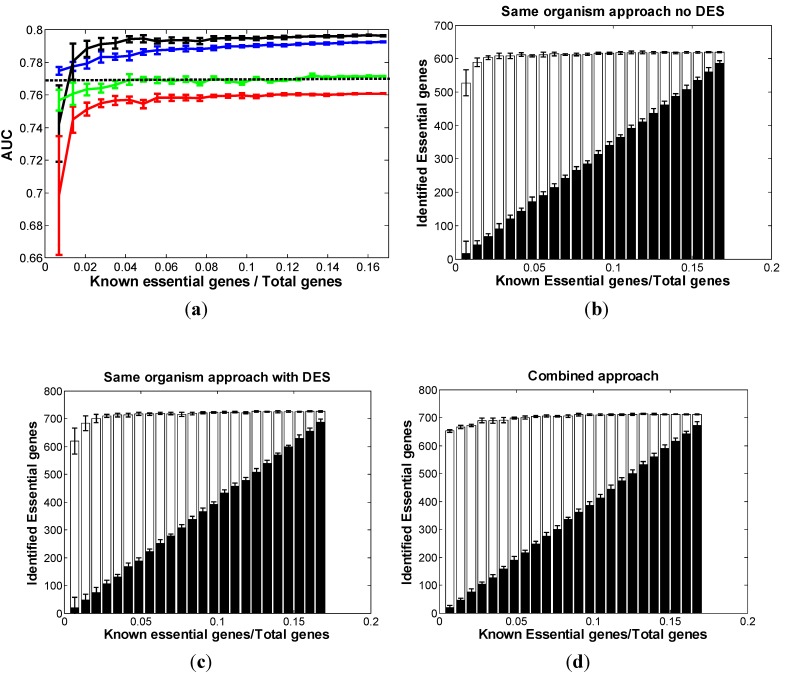
Comparison of three approaches in *NC.* (**a**) The distribution of AUC along with the different sizes of known essential genes in *NC*: red curve: same-organism approach “with no-DES”; black curve: same-organism approach “with DES”; blue curve: combined approach; dashed line: cross-organism approach. The bar chart of the correctly classified essential genes among the top 1500 predictions with respect to the different sizes of known essential genes in *NC* using (**b**) “no-DES” model; (**c**) “with-DES” model; and (**d**) combined model. The black bar shows the correctly classified essential genes in the “gold standard” set.

*NC* is an ascomycete, the red bread mold. Like all fungi, it reproduces by spores. It is used as a eukaryotic model organism because it is easy to grow and has a haploid life cycle which makes genetic analysis easier. There are 1250 essential genes in *NC* produced by the systematic gene deletion project. We identified 14 features potentially associated with gene essentiality in *NC* (Table 1). Following the same procedure as above, we analyzed the “no-DES” and “with-DES” dataset of the same-organism approach separately. We assigned the top 1500 genes as the predicted essential genes. Figure 4a shows that when given about 4% of total genes to be essential, the prediction achieves stable AUC with over 95% best performance. Compared with the red curve, the black curve is significantly improved. The blue curve also showed the performance of the combined approach using *SC* and partial *NC* known essential genes. The conclusion is similar to that in *SC*: The same-organism approach in *NC* (black curve) dominates the combined approach (blue curve) after at least 1.5% of the total genes are known to be essential.

### 3.5. Discussion

Our results suggest that, in prokaryotes, when the number of known essential genes is greater than 2% of total genes, it will achieve over 95% of the best performance, recovering >68% of total essential genes at the given cutoff. For example, for an understudied organism with 3000 genes, we need to know ~60 essential genes in order to accurately predict the majority of its ~300 essential genes. In contrast, in eukaryotes, achieving the same level of performance requires more than 4% of total genes, reflecting the increased complexity of eukaryotic organisms. The complexity comes from different aspects. One possibility is that eukaryotes have more complex genome structures than prokaryotes, such as the expanded protein domain repertoire. In fact, *EC* and *AB* contain 5468 and 4204 unique domains, respectively, while *SC* and *NC* contain 6023 and 7031 unique domains, respectively, according to the Interpro database. In addition, higher organisms have larger and more complex cellular structure as well as perform more diversified functions, which also require them to have more essential genes. 

We found that the required number of known essential genes was surprisingly small for both prokaryotes and eukaryotes, suggesting that the distribution of genomic features extracted from this small subset already provided a close approximation to the distribution of those extracted from the entire essential gene set. This showed the advantage of predicting essential genes using machine-learning approaches. 

**Figure 5:**
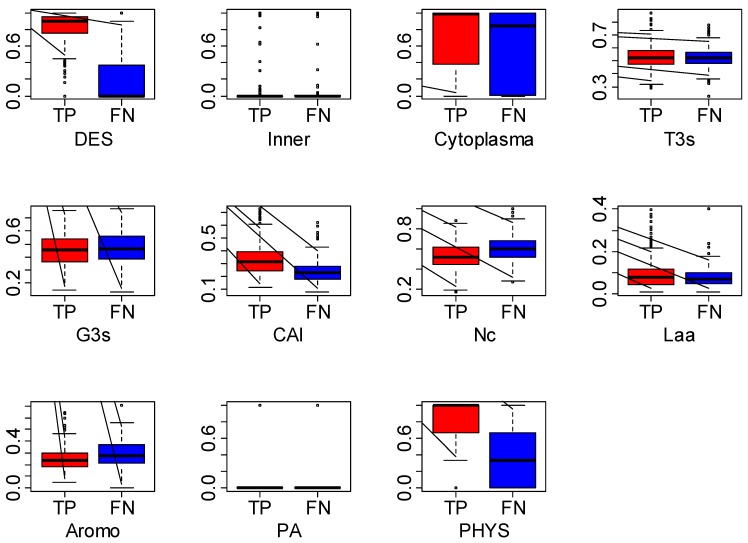
The distribution of features among true positives (TPs) and false negatives (FNs) in *AB*.

We also noticed that as the model reaches saturation, there are still parts of essential genes (*i.e.*, 32% in prokaryotes) that cannot be correctly predicted as essential. We further explored these incorrectly predicted essential genes by plotting the distributions of their associated features. Here we defined the essential genes that were correctly predicted as true positives (TPs) and those that were incorrectly predicted as false negatives (FNs). Figure 5 shows the boxplot of the two parts of genes in *AB*. The features for which the distributions between the two sets of genes differed most widely are DES and PHYS, followed by CAI, Nc and Aromo, all of which were derived from genomic sequences. This suggests that in order to correctly predict the FNs, relying on features based on genomic sequences is no longer enough. Other strong functional genomics features have to be discovered and incorporated into predictions. This observation also supports the notion that gene essentiality is likely determined not solely by genomic sequence, but by multiple aspects of biology, from sequence to function. 

**Figure S1:**
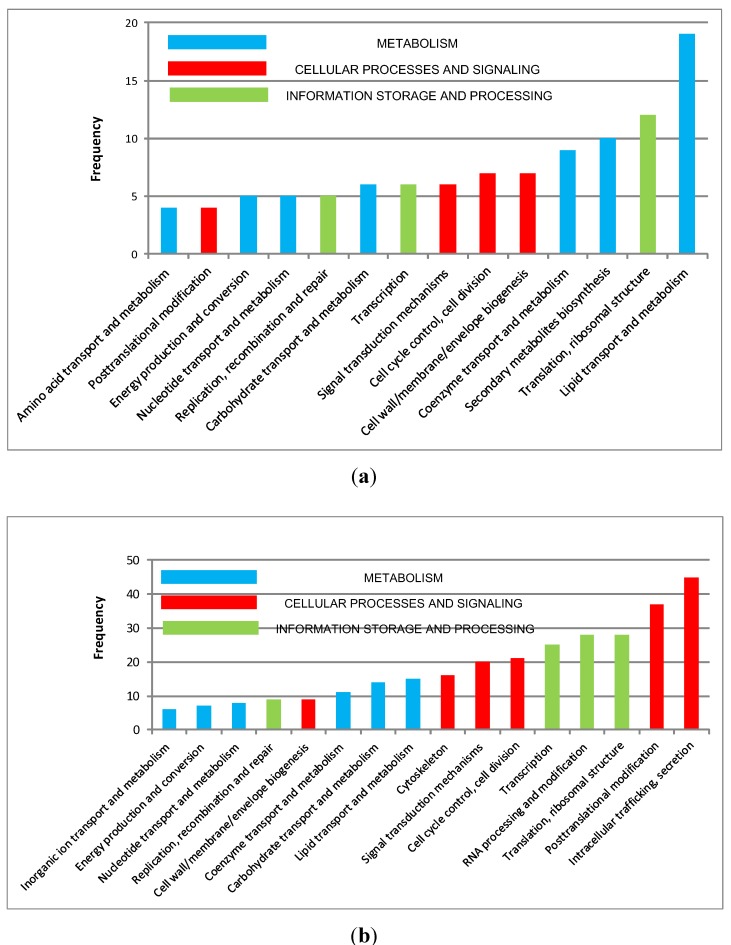
Functional distribution of false negative genes according to the orthologous groups of proteins (COGs) classification in *EC* (**a**) and *SC* (**b**) respectively.

We then performed functional analysis of the FN genes by categorizing them according to the clusters of orthologous groups (COGs) proteins classification. In COGs, genes can be generally classified into four broad functional categories: information storage & processing, cellular processes & signaling, metabolism and poorly characterized. Previous work has shown that essential genes are overrepresented in the category of information storage and processing with basic cellular functions such as RNA processing and modification and DNA replication [32]. Essential genes involved in this category are often well conserved across species. On the other hand, the species-specific essential genes are mainly distributed in cellular processes and metabolic categories, which often reflects a microbe’s unique life style and living environment. Figure S1a and Figure S1b) show the distributions of FN genes across different functional categories in *EC* and *SC* respectively. We can see in *EC* the FN genes are enriched in the metabolic category while in *SC* these FN genes are enriched in cellular processes and signaling category.

Comparing different sets of features used between the prokaryotes (*EC*, *AB*) and eukaryotes (*SC*, *NC*) in Table 1, the common features they shared are: Nc, L_aa, PHYS, PA, DES and FLU. These features cover all three categories described in Section 2.2. This supports our conclusion that the computational integration of different genomic and functional features is able to accurately predict essential genes in both prokaryotes and eukaryotes. However, there are some differences of features used between them, such as those sub-cellular localization features. For example, Nucleus, Plasma and PredHel are used only by *SC* and *NC* while Inner member is used only by *EC* and *AB*. These reflect the differences in cellular structure between prokaryotes and eukaryotes—the eukaryotic cells are much larger and more complex than prokaryotic cells.

Through our analysis, we realize that the evolutionary distance between the understudied organism and the model organism may affect the thresholds observed in our study. Nevertheless, our results suggest that an organism’s own known essential genes usually contain more information about its unique physiology and are a better representative set of its total essential genes.

Logistic regression was chosen in this study mainly because of its simplicity and ease of interpretation of results. Other machine-learning methods could have been used. However, most alternative techniques suffer from their own limitations, e.g., missing value problems or being prohibitively time-consuming, which prevent them from being used in this study. Nevertheless, we expect our conclusions from this investigation are unlikely to change if a different machine-learning technique is used. Since the four species we studied are all microorganisms, the conclusions from this study may not be applicable to more complex systems, such as mouse and human. Finally we believe the results obtained from our study provided important information on accurately predicting essential genes and will greatly facilitate the annotations of microbial genomes. 

## 4.Conclusion

In this study, we investigated the performance of three approaches for predicting essential genes under conditions where information on different numbers of known essential genes is given. Our results suggest that when determining the best strategy for predicting essential genes, unless the number of known essential genes is small, *i.e.*, less than 1.5% of total genes, learning from the known essential genes in the target organism usually outperforms transferring essential gene annotations from a related model organism. This is consistent in both prokaryotes and eukaryotes. Moreover, when the known essential genes are few (*i.e.*, <1.5% of total genes), and a closely related organism is available, combining these two sources of information results in a significantly increased performance over either the same-organism approach or the cross-organism approach. On the other hand, when the target organism has a sufficiently large number of known essential genes, combining the annotations from a model organism often results in a reduced performance as compared with using its own known essential genes, reflecting the slight differences of the underlying properties of essential genes between different organisms.

## Supplementary Section

### Intrinsic and Context-Dependent Genomic Features

To create a training dataset for our classifier, features are extracted where available for each ORF in each organism and annotated with known essentiality values from the essential gene datasets. Our study considers three main types of features: (A) those intrinsic to a gene’s sequence (e.g., GC content, length); (B) those derived from genomic sequence (e.g., localization signals and codon adaptation measures) and (C) experimental functional genomics data (e.g., gene-expression microarray data).

A-1. *Genomic sequence properties*: We use CodonW (http://bioweb.pasteur.fr/seqanal/interfaces/codonw.html) to calculate the following properties associated with genomic sequences: Kyte and Doolittle’s grand average of hydropathicity (GRAVY) [1], protein length (amino acids), GC content, and two measures of codon usage: effective Nc [2,3] and CAI [4].

B-1. *Predicted subcellular localization*: We used the PA-SUB Server v2.5 to obtain these features [5]. Gram-negative bacteria (*EC*, *PA* and *AB*) have five predicted localizations: Inner membrane, Extracellular, Cytoplasm, Periplasm, Outer membrane. Gram-positive bacteria (*BS*) have three predicted localizations: Extracellular, Cytoplasm, Plasma membrane.

B-2. *Transmembrane helices for* each *ORF*: The putative transmembrane helices are calculated by TMHMM Web server v2.0 [6,7].

B-3. *Evolutionary conservation of a gene*: We used the RBH method to search orthologs in multiple complete genomes for each gene of the target organism (*PA*, *EC*, *AB* and *BS*). The number of genomes that have orthologous hits was used as a measure of evolutionary conservation of a gene. Such conservation has been shown to correlate well with the dispensability of a gene [8].

B-4. *Paralogy*: Duplicated genes in an organism are often referred to as paralogs. An all-against-all FASTA search was conducted for the whole set of ORFs in the target organism (*PA*, *EC*, *AB* and *BS*) to identify the paralogs with an E-value threshold of 10^−20^.

B-5 *Domain enrichment*: For each individual domain, we collected its occurrence in each organism (*PA*, *EC*, *AB* and *BS*) using the Pfam database (http://pfam.sanger.ac.uk). Then we estimated the domain enrichment score according to the ratio of occurrence frequencies between essential gene sets and the total genes in the target organism: , here n_ess_ and n_non-ess_ represent a domain’s occurrence frequency in the essential and non-essential dataset, respectively. N_ess_ and N_non-ess_ is the size of the essential and non-essential dataset, respectively.

C-1. *Fluctuation in gene-expression*: The mRNA expression levels of essential genes often vary, on average, within a narrower range, whereas the expression of nonessential genes fluctuates more widely [9]. Gene expression data in these bacteria were downloaded from NCBI GEO [10], ArrayExpress [11], as well as the gene-expression profiles of microarray data from Gasch *et al*. [12]. The variance of each gene was calculated from these gene expression profiles as a measure of the fluctuation of gene expression.

C-2. *Topology in gene co-expression network*: From gene expression microarray data, a gene-expression cooperativity graph is constructed as *G_g_*(*D*) = (*V_g_,E_g_*), with the vertex set  and the edge set  for  and | *r_ij_* | ≥ 0.7. Each vertex represents a gene and each edge represents a gene pair whose gene expression profiles correlation coefficient | *r_ij_* | is greater than 0.7. This cutoff value of | *r_ij_* | is determined based on our previous work [13]. The hubs (nodes with high degrees) and bottlenecks (nodes with high betweenness or shortest paths occurrence) have been found to have correlations with gene essentiality [14]. The network statistics are calculated using tYNA (http://www.gersteinlab.org/tyna).
